# Dietary supplementation with *Moringa oleifera* leaves extract reduces the impacts of sub-lethal fipronil in Nile tilapia, *Oreochromis niloticus*

**DOI:** 10.1038/s41598-022-25611-6

**Published:** 2022-12-16

**Authors:** Hemat K. Mahmoud, Mayada R. Farag, Fayiz M. Reda, Mahmoud Alagawany, Hany M. R. Abdel-Latif

**Affiliations:** 1 Department of Animal Production, Faculty of Agriculture, Zagazig University, Zagazig, 44511 Egypt; 2 Forensic Medicine and Toxicology Department, Veterinary Medicine Faculty, Zagazig University, Zagazig, 44511 Egypt; 3 Poultry Department, Faculty of Agriculture, Zagazig University, Zagazig, 44511 Egypt; 4 Department of Poultry and Fish Diseases, Faculty of Veterinary Medicine, Alexandria University, Alexandria, Egypt

**Keywords:** Zoology, Natural hazards

## Abstract

This study assessed the restorative dietary effects of *Moringa oleifera* (MO) leaves extract against the negative impacts of sub-lethal fipronil (FIP) toxicity in Nile tilapia. To achieve this purpose, the growth, body composition, haemato-biochemical measurements, serum immunity, and antioxidant condition of Nile tilapia have been examined. Fish were arranged into 6 experimental groups in quadruplicates. Three groups were fed on diets supplemented with 0.0 (reference group), 1.0 (MO1), and 2.0 (MO2) g kg^−1^ of MO leaf extract. The other three groups were fed on the same MO levels and concomitantly subjected to a sub-lethal FIP concentration (4.2 µg L^−1^ for 3 h only per day) and defined as FIP, FIP + MO1, and FIP + MO2. The experiment lasted for 8 weeks. Results unveiled that growth parameters were significantly decreased alongside an increased feed conversion ratio in the FIP-intoxicated group. The moisture and crude protein (%) were decreased significantly together with a significant increase of the crude lipids (%) in the fish body of the FIP group. Sub-lethal FIP toxicity induced hypochromic anemia, leukopenia, hypoproteinemia, hypoalbuminemia, hypoglobulinemia, and hepato-renal failure (increased urea and creatinine concentrations, as well as ALT and AST enzymes). Exposure to sub-lethal FIP also induced (a) immunosuppression manifested by a decline in total IgM, complement C3, and lysozyme activities, (b) enzymatic antioxidant misbalance manifested by decreases in SOD and CAT activities, and (c) oxidative stress (declined T-AOC and elevated of MDA concentrations). On the other side, dietary supplementation with MO leaf extract in FIP + MO1 and FIP + MO2 groups noticeably modulated the aforementioned parameters. Therefore, we can conclude that dietary MO could reduce sub-lethal FIP toxicity in Nile tilapia with a possible recommendation for regular prophylaxis supplementation in Nile tilapia diets.

## Introduction

*Moringa oleifera* (MO) is known as the “Miracle tree” and is mostly available in several tropical and sub-tropical countries throughout the world^1^. It has several therapeutic and health benefits due to its anti-inflammatory, hepatoprotection, antimicrobial, and antioxidant effects^2–6^. Its leaves contain high crude protein levels and essential amino acids^7,8^. The leaves are also abundant in several essential vitamins, minerals (like potassium, iron, and calcium)^9^, and phytochemicals (like carotenoids, flavonoids, phenolics, alkaloids, and terpenoids^10^. Due to the above-mentioned nutritive values, studies elucidated that MO-based meals could be proficiently consumed by poultry^11^, goats, cattle^12,13^, and fish^14,15^. The presence of anti-nutrient (anti-nutritional) factors may hinder the digestibility of MO-based diets, and in this case, it requires several processing techniques^14^. In aquaculture studies, it has been found that MO-supplied diets could enhance the growth of rohu carp^16^ and Nile tilapia^17^, nutrient digestibility of *Pangasius bocourti*^18^, strengthen the antioxidant ability^19^, and attenuate starvation stress^17^. MO leaf extract could also potentiate innate immune responses and increase the defense against the challenged pathogenic agents^20^.

Fipronil (FIP) is classified as a phenylpyrazole insecticide that is active against various insects and pests that infest agriculture crops^21,22^. Its insecticidal effects were performed through binding to γ-aminobutyric acid (GABA) receptors and playing as a glutamate-gated chloride (Glu-Cl) and GABA-gated chloride (GABA-gated-Cl) channels in the CNS of insects producing neuronal paralysis, hyperexcitability, and insect death^23^. This insecticide has been detected in the aquatic environments between 0.5 and 9 μg L^−1^ in the treated rice fields water surfaces^24^ and up to 12.6 μg L^−1^ in urban domestic runoff^25^. The continuous use and unhygienic disposal of this hazardous material will lead to its disposition in the aquatic ecosystems and subsequently induce serious risk to the exposed fish^26^.

Studies showed that fish exposure to FIP, even at the environmentally relevant levels, induced hazardous toxic effects. For instance, sub-lethal FIP dose (0.65 μg L^−1^) induced oxidative stress via alteration of the antioxidant enzyme system of common carp^27^. The exposure of *Prochilodus lineatus* to environmentally relevant levels of FIP provoked oxidative stress in the exposed gills^28^. FIP exposure also caused histopathological lesions and immune suppression in Nile tilapia^29,30^, impaired embryonic development of Japanese Medaka^31^, and hematological, genotoxic, and histopathological effects of common carp^32,33^. FIP also induced hepato-renal failure and histopathological damage of rohu carp^34^, deterioration of the metabolic and physiological biomarkers of European seabass^35^, and oxidative stress damage and genotoxic effects in rainbow trout^36,37^.

Some reports showed that MO could modulate the sub-lethal toxic impacts of several toxicants, such as CuONPs in common carp^38^ and hydrogen peroxide in gilthead seabream^39^. Reports published in Nile tilapia showed that dietary MO could ameliorate and reduces the sub-lethal toxic effects of several toxicants such as chlorpyrifos^40^, pendimethalin^41^, and aflatoxin B1 (AFB1)^42^. However, the literature on the potential efficacy of MO leaves extract as a feed supplement to lessen or mitigate the sub-lethal toxic effects of FIP is scarce. Thus, this study was planned to assess the long-term sub-lethal toxic impacts of FIP on stress factors, growth, water quality, chemical analysis of the fish body, serum biochemistry (particularly hepato-renal bioindicators and lipid profile), immunity, and antioxidant state of Nile tilapia. Moreover, the attenuating effects of dietary MO leaf extract were also examined.

## Materials and methods

### Ethical statement

All experimental works in the present study were performed following the Local Experimental Animal Care Committee guidelines and approved by the Institutional Ethics Committee of the Faculty of Agriculture, Zagazig University, Egypt and all methods were performed in accordance with the relevant guidelines and regulations (Approval number: ZUIACUC/2/F/110/2022).

### Fipronil

Fipronil (FIP) technical grade (97%) is commercially purchased from Jiangsu Tuoqiu Agrochemicals Co., Ltd. (China) (CAS Number: 120068-37-3). It was used to prepare the stock solution immediately before the exposure.

### MO leaf extract

Fresh MO leaves were acquired from the local market, Sharkia governorate, Egypt. The ethanolic extract of MO leaf was made following the protocol illustrated in^43^. Moringa leaves were washed thoroughly using distilled water (DW) and then permitted to dry at room temperature (30–34 °C) for 21 days. Next, dried moringa leaves were then finely milled by a high-speed milling machine. Subsequently, the dried MO leaf powder (1 kg) was extracted in one liter of 100% ethanol for 2 days and then filtered two times using a filter paper with a 2-μm sized pore. The ethanolic extract was condensed at 50 °C by means of a rotary evaporator. MO leaf ethanolic extract yields (78.3 g kg^−1^ of the dried MO leaf powder). The procured MO ethanolic extract was resuspended (1 g of MO leaf extract in 10 mL DW) and was then kept at 4 °C in the refrigerator.

### Fish acclimation, rearing, and management practices

Nile tilapia (14.50 ± 0.50 g) were transported to the Wet Fish Laboratory, Department of Animal Production, Faculty of Agriculture, Zagazig University, Egypt. Fish were conditioned for 12 days in glass aquaria supplied with chlorine-free tap water (stored for 48 h before receiving fish). Rearing water was provided with compressed air by a central air compressor. Fish were hand-fed with a well-balanced basal diet (contains 30% CP) (commercially purchased from Aller aqua Co., Egypt). Table S1 (supplementary materials) illustrates the nutritional elements found in this diet and its analytical constituents. Fish were fed on this diet 3 times daily (till noticeable satiation). The light schedule is maintained as 12 h light and 12 h dark. During the acclimation and experimental periods, about 35% of the rearing water was syphoned off daily to eliminate the non-eaten feed particles and faeces and later replaced with other water from the storage tanks.

### Experimental design

Two hundred and forty Nile tilapia were allocated into 24 glass aquaria (35 cm × 40 cm × 70 cm) (85 L each) that were arranged into 6 groups (in quadruplicates), and each replicate holds 10 fish. Three groups were fed on prepared diets supplied with freshly prepared MO ethanolic leaf extract (0.0, 1.0, and 2.0 g kg^−1^ diet) and defined as control (CTR), MO1, and MO2 groups. The other three groups were fed diets provided with the same MO levels (0.0, 1.0, and 2.0 g kg^−1^ diet) and concomitantly subjected to a sub-lethal FIP dose (4.2 µg L^−1^ for 3 h only per day) and defined as FIP, FIP + MO1, and FIP + MO2 groups. The experiment has lasted for 8 weeks.

### FIP exposure and water siphoning regimen

The FIP exposure dose was chosen in line with the previously performed studies on sub-lethal toxic levels of FIP in the same fish species^29,30^. This exposure dose was below the environmentally relevant levels of FIP in the aquatic environments^24,28^. More importantly, Zhao^44^ reviewed that in the studies where FIP will be exposed to light, its half-life will be 3.6 h in water. Therefore, the experiment was designed to allow fish exposure to a sub-lethal FIP concentration in FIP, FIP + MO1, and FIP + MO2 groups only for 3 h daily to avoid FIP degradation and to maintain its required exposure concentration. Water was siphoned daily 3 h post-exposure in FIP, FIP + MO1, and FIP + MO2 groups and replaced with new water. On the other side, water siphoning was also performed daily in the non-intoxicated groups (CTR, MO1, and MO2 groups), whereas water was exchanged with new well-aerated water from the storage tanks. The study was conducted following ARRIVE guidelines^45^.

### Water quality measurements

The water measurements, such as water temperature and pH values, have been examined every week by AD8000 Professional Multi-Parameter pH-ORP-Conductivity-TDS-TEMP Bench Meter. Dissolved oxygen (DO) was evaluated by AD130 Proof PH-ORP-TEMP Portable Meter ADWA (Adwa Instruments, Inc. 6726 Szeged, Alsó-Kikötő sor 11.C, Hungary). Total ammonia nitrogen (TAN) was measured by Hach Kit model HI 83205 (Multiparameter Bench Photometer, Hanna Instruments, and Romania), and then the unionized ammonia (NH_3_) levels were assessed.

### Growth parameters and equations

Fish (in each group) was individually weighed to evaluate the growth and feed efficiency indexes following the calculations published in^46–48^.

$${ {\text{WG}}}\;{{\text{(g)}}} = {{\text{ FBW }}} - {{\text{IBW}}}$$

$${{\text{FI}}}\;( {{{\text{g }}}\;{{\text{feed/fish}}}} ) = {{\text{ The total amount of the feed eaten by fish in the experiment by their total number}}}$$

$${\text{FCR }} = {\text{ FI/WG}}$$

$${{\text{SGR}}}\; ( {\% /{\text{day}}} ) = [ {( {{{\text{Ln FBW }}} - {{\text{Ln IBW}}}} )/{\text{T}}} ] \, \times \, 100$$

$${{\text{SR}}}\;{{{(\% ) }}} = 100 \, \times \, ( {{{\text{The fish number at the end of the experiment/their initial number}}}} )$$

WG = weight gain, FBW: final body weight, IBW: initial body weight, FI = feed intake, FCR = feed conversion ratio, SGR = specific growth rate, SR = survival rate, and T = feeding period.

### Analysis of the fish body composition

The methods used to evaluate the chemical constituents such as crude protein (CP), crude fiber (CF), crude lipids (CL), ash, and moisture of the whole body of the fish, as well as the basal diet, were evaluated^49^. Moisture (%) was measured by oven-drying of the fish samples (at 110 °C) to constant weight (JSON-100, Gongju-City, Republic of Korea). The CP (%) was assessed by the procedures described in the Kjeldahl Distillation Unit (UDK 129, VelpScientifica, Usmate Velate, Italy). The CL (%) was obtained by the Soxhlet extraction method. Moreover, the ash (%) was measured by incineration of the samples in a Muffle furnace at high temperatures (550 °C) for 6 h (Barnstead/Thermolyne Benchtop 47900, Thermo Scientific, Massachusetts, USA).

### Blood sampling and serum collection

Fish were prevented from feeding for one day before sampling of blood. Three samples were collected from 3 fish per replicate (12 fish/group). After the fish were anesthetized by MS-222, the blood was sampled from the fish caudal veins using disposable sterile syringes. Blood samples were apportioned into two parts; one was assembled with heparin (as an anticoagulant) for hematological indexes. The second part was left at room temperature for the collection of the serum. Blood samples were centrifuged at 3500×*g* for 20 min. The collected serum was then preserved in the refrigerator at − 20 °C.

### Haemato-biochemical indices

Erythrocytes and leukocyte counts were deliberated by a hemocytometer. Hemoglobin (Hb) concentrations were calculated in accordance with Collier^50^. Serum samples were examined for the blood proteins such as total protein (TP) and albumin (ALB) values which were established using specific diagnostic kits (Bio diagnostic Co., Dokki, Giza, Egypt). Globulin (GLO) concentrations were evaluated by subtracting ALB from TP concentrations. Kidney function markers such as blood urea nitrogen, creatinine, and uric acid levels were also measured. Liver enzymes such as alanine transaminase (ALT), lactate dehydrogenase (LDH), and aspartate transaminase (AST) activities were carried out colorimetrically using specific diagnostic kits (Bio diagnostic Co., Dokki, Giza, Egypt). Serum lipid profile including triglycerides (TG), very-low-density lipoprotein (VLDL-c), total cholesterol (TC), high-density lipoprotein (HDL-c), and low-density lipoprotein (LDL-c) was evaluated Bio diagnostic kits (Bio diagnostic Co., Dokki, Giza, Egypt).

### Immunity biomarkers

The fish serum lysozyme (LZ) activities were evaluated by the turbidimetric assay by freeze-dried *Micrococcus lysodeikticus* (Sigma-Aldrich, St. Louis, MO) as a substrate^51^. One unit of the serum LY activity was outlined as the decline of 0.001 of optical density OD_520_ /min. The fish complement C3 contents were measured by quantitative sandwich ELISA by utilizing special fish kits (named the Fish Complement Component 3) (Catalog #MBS005953) (MyBioSource, Inc. San Diego, CA, USA). The total immunoglobulin M (IgM) levels were measured using ELISA kits. Serum nitric oxide (NO) levels were assessed spectrophotometrically by commercial kits (BioChain, Inc., USA) (in line with the technical information obtained from the manufacturer).

### Antioxidant bioindicators

Catalase (CAT) enzyme, superoxide dismutase (SOD) enzyme, and reduced glutathione (GSH) levels were measured colorimetrically by specific diagnostic kits (Bio diagnostic Co., Giza, Egypt). Serum malondialdehyde (MDA) contents were colorimetrically measured by a commercial kit that measured free and total MDA compounds (LPO, OXIS Int., USA). Total antioxidant capacity (T-AOC) is evaluated in fish serum using specific kits (Bio diagnostic Co., Giza, Egypt). One unit of T-AOC was expressed as the amount of sample produced an increase in absorbance of 0.001 per min.

### Statistical assessment

The data in the excel sheets were examined by one-way ANOVA. Tukey's test was done as a post-hoc test to evaluate the differences between the means in each experimental group, whereas P < 0.05 was statistically significant. Analyses were achieved by SPSS program 22.

## Results

### Water quality parameters

The weekly mean values of water quality parameters in the rearing aquaria in which fish were fed diets supplemented with MO leaf extract and/or intoxicated with sub-lethal FIP dose for 8 weeks were described in Table 1. Results showed that dietary supplementation with MO leaf extract significantly (P < 0.05) decreased the un-ionized ammonia and increased the DO levels. The opposite trend was observed in all FIP-intoxicated groups. The lowest DO and the uppermost unionized ammonia levels were noticed in the FIP group. Interestingly, MO groups exposed to FIP showed improved DO and ammonia levels than FIP only. Water temperature and pH values were not significantly influenced (P > 0.05; Table 1).

**Table 1:** Water quality parameters in the rearing aquaria of Nile tilapia groups during the present study.

Items	Experimental groups						SEM	*P* value
	CONT	MO1	MO2	FIP	FIP + MO1	FIP + MO2		
Temperature (°C)	30.07	29.93	29.9	29.95	29.97	29.93	0.185	0.9916
Dissolved oxygen (mg L^−1^)	6.00 bc	6.08 ab	6.23 a	5.29 e	5.68 d	5.83 cd	0.062	< 0.0001
pH values	8.12	8.19	8.16	8.03	8.14	8.12	0.219	0.9974
Un-ionized ammonia (mg L^−1^)	0.037 cd	0.028 d	0.032 d	0.094 a	0.062 b	0.045 c	0.003	< 0.0001

Data represent means ± S.E.M—means having different letters in the same row are significantly different at *P* < 0.05.

### Growth and feed utilization parameters

Growth, feed utilization, and SR of fish fed diets supplied with MO leaf extract and/or intoxicated with sub-lethal FIP dose for 8 weeks were illustrated in Table 2. Daily feed intake, total feed intake, body mass gain, FBW, WG, and SGR were increased (P < 0.05) significantly in all MO groups, and their uppermost values were found in the MO2 group. FCR levels decreased significantly (P < 0.05) in all MO groups, and their lowest values were found in the MO2 group. On the other hand, all growth and feed utilization indexes were significantly (P < 0.05) decreased, and FCR values were (P < 0.05) increased in the FIP-intoxicated group, suggesting the occurrence of growth depression in this group. However, dietary supplementation with MO (FIP + MO1 and FIP + MO2 groups) significantly modulated the parameters mentioned above in comparison with the FIP group (Table 2). SR (%) was lower in the FIP group than in other experimental groups.

**Table 2:** Growth performance, feed utilization indices and survival rates of Nile tilapia groups during the present study.

Items	Experimental groups						SEM	*P* value
	CONT	MO1	MO2	FIP	FIP + MO1	FIP + MO2		
Initial body weight (g)	14.82	14.87	14.87	14.90	14.83	14.85	0.159	0.9992
Final body weight (g)	53.71 c	58.11 b	61.68 a	40.21 e	50.42 d	51.44 d	0.668	< 0.0001
Weight gain (g)	38.89 c	43.23 b	46.82 a	25.31 e	35.59 d	36.59 d	0.637	< 0.0001
Specific growth rate (%/day)	1.53 c	1.62 b	1.69 a	1.18 e	1.46 d	1.48 d	0.015	< 0.0001
Body mass gain (%)	262.54 c	290.66 b	315.00 a	169.91 e	240.08 d	246.46 d	4.702	< 0.0001
Daily feed intake (g)	0.95 b	1.02 a	1.05 a	0.86 d	0.91 c	0.92 bc	0.011	< 0.0001
Total feed intake (g)	79.99 b	85.36 a	88.19 a	72.38 d	76.23 c	77.38 bc	0.911	< 0.0001
Feed conversion ratio	2.06 bc	1.97 cd	1.88 d	2.86 a	2.14 b	2.12 b	0.030	< 0.0001
Survival rate (%)	98.33 a	98.33 a	96.67 a	80.00 b	93.33 a	95.00 a	2.351	0.0016

Data represent means ± S.E.M—means having different letters in the same row are significantly different at *P* < 0.05.

### Proximate body composition

Moisture and CP (%) were significantly (P < 0.05) elevated, combined with a significant (P < 0.05) decline of the CL (%) in MO groups compared to other groups (Table 3). The opposite trend was observed in the FIP group. However, significant modulation of these parameters was noticed in groups fed MO diets and consequently exposed to FIP. However, no differences were found in the ash (%) among all groups (Table 3).

**Table 3:** Whole body composition (% on dry weight basis) of Nile tilapia groups during the present study.

Items	Experimental groups						SEM	*P* value
	CONT	MO1	MO2	FIP	FIP + MO1	FIP + MO2		
Moisture (%)	75.01 bc	76.70 a	76.36 ab	73.75 c	75.56 bc	75.40 bc	0.434	0.0042
Crude protein (%)	56.02 bc	58.11 a	58.28 a	55.04 c	56.99 ab	57.30 ab	0.494	0.0128
Crude lipids (%)	17.21 bc	17.80 b	17.96 b	18.89 a	16.89 c	17.98 b	0.221	0.0011
Ash (%)	17.58	17.91	17.48	17.73	18.67	17.55	0.590	0.7819

Data represent means ± S.E.M—means having different letters in the same row are significantly different at *P* < 0.05.

### Haemato-biochemical indices

In comparison with the CTR group, MO leaves extract did not significantly alter erythrocyte count, Hb, TP, ALB, ALT, AST, creatinine, and blood urea nitrogen levels except for a significant increase in leukocyte count and GLO levels (Table 4). Conversely, Hb levels, erythrocytes, leukocyte count, TP, ALB, and GLO values were the lowest in the FIP group. Furthermore, ALT, AST, blood urea nitrogen, and creatinine levels were the highest in the FIP group, and these results indicated the occurrence of hepato-renal damage. Interestingly, dietary supplementation with MO leaf ethanolic extract in FIP-intoxicated groups significantly modulated these parameters compared with the FIP-intoxicated group alone without moringa supplementation. Oppositely, no statistical differences were recorded in LDH and uric acid levels among all groups (P > 0.05; Table 4). Regarding the lipid profile of treated fish, it was noticed that HDL-c, TG, LDL-c, TC, and VLDL-c levels were decreased significantly in both MO groups (P < 0.05; Table 5). However, the reverse trend was observed in the FIP-intoxicated group, which indicates hypercholesteremia, hypertriglyceridemia, and disturbance of lipid metabolism in the FIP group. However, significant modulations of these indices were noticed in the FIP + MO1 and FIP + MO2 groups compared to FIP alone (Table 5).

**Table 4:** Haemato-biochemical measurements of Nile tilapia groups during the present study.

Items	Experimental groups						SEM	*P* value
	CONT	MO1	MO2	FIP	FIP + MO1	FIP + MO2		
Hemoglobin (g dL^−1^)	7.99 a	7.70 a	7.98 a	5.13 b	7.33 a	7.36 a	0.259	0.0002
Erythrocytes (10^6^ × µL)	3.38 a	3.57 a	3.50 a	2.83 c	3.06 bc	3.30 ab	0.096	0.0014
Leukocytes (10^3^ × µL)	8.65 b	10.07 ab	11.32 a	6.19 c	9.61 b	8.69 b	0.461	0.0002
Total protein (g dL^−1^)	5.63 a	5.62 a	5.78 a	4.00 b	5.61 a	5.36 a	0.121	< 0.0001
Albumin (g dL^−1^)	3.17 a	3.02 a	3.06 a	2.17 b	3.24 a	3.18 a	0.084	< 0.0001
Globulin (g dL^−1^)	2.46 bc	2.60 ab	2.72 a	1.82 e	2.37 cd	2.18 d	0.062	< 0.0001
Alanine aminotransferase (U L^−1^)	28.39 d	28.67 d	25.40 d	53.89 a	44.83 b	36.94 c	1.799	< 0.0001
Aspartate aminotransferase (U L^−1^)	88.87 c	76.30 c	83.17 c	140.02 a	116.50 b	125.67 b	4.440	< 0.0001
Lactate dehydrogenase (U L^−1^)	31.85	30.9	36.43	40.47	35.73	33.60	4.116	0.6605
Creatinine (mg dL^−1^)	0.82 c	0.96 c	0.86 c	1.39 a	1.15 b	1.16 b	0.051	< 0.0001
Urea (mg dL^−1^)	7.82 c	7.23 c	7.50 c	11.18 a	10.97 b	10.29 b	0.563	0.0012
Uric acid (mg dL^−1^)	11.33	10.93	10.77	14.09	11.81	12.03	0.829	0.1476

Data represent means ± S.E.M and means having different letters in the same row are significantly different at *P* < 0.05.

**Table 5:** Lipid profile of Nile tilapia groups during the present study.

Items	Experimental groups						SEM	*P* value
	CONT	MO1	MO2	FIP	FIP + MO1	FIP + MO2		
Total cholesterol (mg dL^−1^)	188.52 b	153.42 d	158.47 cd	213.28 a	188.64 b	176.73 bc	6.404	0.0004
Triglycerides (mg dL^−1^)	181.67 b	134.27 c	141.27 c	239.20 a	176.93 b	167.87 b	5.644	< 0.0001
HDL-c (mg dL^−1^)	49.88 b	50.23 b	36.83 c	64.43 a	48.72 b	51.46 b	2.210	< 0.0001
LDL-c (mg dL^−1^)	102.31 b	65.33 c	65.79 c	128.61 a	104.54 b	91.70 b	4.706	< 0.0001
VLDL-c (mg dL^−1^)	36.33 b	26.85 c	28.25 c	47.84 a	35.39 b	33.57 b	1.128	< 0.0001

Data represent means ± S.E.M—means having different letters in the same row are significantly different at *P* < 0.05.

*HDL-c* high density lipoprotein, *LDL-c* low density lipoprotein, *VLDL-c* very low-density lipoprotein.

### Serum immunological assays

Serum immune assays of Nile tilapia fed diets provided with MO leaf extract and/or intoxicated with sub-lethal FIP level for 8 weeks were shown in Table 6. Total IgM, complement C3, and LYZ levels (P < 0.05) increased significantly in all MO groups than the FIP and CTR groups. Contrariwise, their levels were the lowest in the FIP-intoxicated group, suggesting the immune-suppressive effects of FIP intoxication.

**Table 6:** Serum immune and antioxidant biomarkers of Nile tilapia groups during the present study.

Items	Experimental groups						SEM	*P* value
	CONT	MO1	MO2	FIP	FIP + MO1	FIP + MO2		
Immunoglobulin M (ng mL^−1^)	0.60 b	0.77 a	0.89 a	0.40 c	0.85 a	0.77 a	0.067	0.0056
Complement C3 (mg dL^−1^)	9.97 c	12.43 b	14.18 a	6.89 d	12.70 ab	11.47 bc	0.468	< 0.0001
Lysozyme (ng mL^−1^)	1.70 bc	3.15 a	2.83 a	1.35 c	1.85 b	1.85 b	0.135	< 0.0001
Superoxide dismutase (U mL^−1^)	3.23 ab	3.50 a	3.45 a	2.18 c	3.07 b	2.95 b	0.102	< 0.0001
Catalase (U mL^−1^)	7.85 abc	8.48 ab	8.72 a	6.72 c	7.41 bc	7.64 abc	0.345	0.0190
Malondialdehyde (nmol mL^−1^)	5.62 c	4.77 c	4.87 c	12.46 a	7.46 b	7.91 b	0.408	< 0.0001
Reduced glutathione (nmol mL^−1^)	1.60	1.64	1.70	1.50	1.51	1.56	0.088	0.6088
Total antioxidant capacity (ng mL^−1^)	1.51 b	1.82 a	1.87 a	1.20 c	1.49 b	1.60 b	0.062	< 0.0001
Nitric oxide (nmol mL^−1^)	1.39 b	1.22 bc	1.05 c	1.67 a	1.30 bc	1.22 bc	0.053	0.0030

Data represent means ± S.E.M—means having different letters in the same row are significantly different at *P* < 0.05.

### Antioxidant biomarkers

Serum antioxidant biomarkers of Nile tilapia that fed diets supplied with MO leaf extract and/or intoxicated with sub-lethal FIP dose for 8 weeks were shown in Table 6. It was found that antioxidant enzyme activities (SOD and CAT activities), NO values, and T-AOC levels were significantly increased, combined with a decline (P < 0.05) of MDA levels in MO-supplied groups than the FIP group. These positive effects of dietary MO suggest the potent antioxidant potential of dietary MO. The reverse trend was observed in the FIP group, which indicates the oxidative stress injury of FIP toxicity. Attractively, FIP + MO1 and FIP + MO2 groups showed significant attenuation of these parameters compared with FIP alone. However, no significant differences were observed in the GSH levels amongst experimental groups (P > 0.05; Table 6).

## Discussion

Fipronil (FIP) is a highly effective insecticide against a wide range of agriculture pests^22^. Its disposition in the aquatic ecosystems will induce hazardous effects on the exposed aquatic organisms^26^. Literature indicated that exposure to environmentally relevant levels of FIP could induce fish toxicity signs. Herein, we have evaluated the long-term sub-lethal toxicity of FIP in Nile tilapia. Also, we assessed the possible attenuating effects of dietary MO leaf ethanolic extract. We found improved water parameters in tanks, whereas fish was reared on MO leaf extract-supplemented diets compared to the FIP-intoxicated group. These findings were similar to those obtained by^52^, who found that the water quality measurements were improved in tanks where Nile tilapia was reared on diets supplied with a powder prepared from MO leaves for two months. These results might be accredited to the reduced nutrient load in the water, consequently leading to a decreased deterioration of water and accumulation of toxicants^53^. The deteriorated physicochemical parameters of water in which fish were exposed to pesticides may be because of increased organic material in the water, as declared by Sweilum^54^. Nonetheless, the precise mechanisms by which MO attenuated water parameters are unclear and necessitate further studies.

The growth and feed utilization parameters were improved in MO groups in relation to the FIP group. Moreover, growth indices were improved considerably upon combined dietary supplementation with MO leaf extract with FIP exposure. These findings agreed with those declared by Elabd et al.^17^, who found that 1.5% MO leaf meal considerably improved Nile tilapia growth after three months of feeding experiment. Those authors also found that MO meal efficiently mitigated the growth performance after starvation stress. Another study demonstrated by^52^ illustrated that supplementing diets with MO leaves powder increased monosex tilapia growth after 2 months of the feeding experiment. Dietary 7% MO seeds or 10% MO leaves substantially normalized the growth performance to the control values in chlorpyrifos-intoxicated Nile tilapia^40^. Abdelhiee et al.^42^ recently showed that 0.5% MO seed extract efficiently enhanced the growth performance of Nile tilapia exposed to a sub-lethal AFB1 level. Several studies also demonstrate the efficacy of some natural supplements to lessen the toxic effects of FIP on the fish growth. For instance, e.g., vitamin C modulated the growth performance of common carp intoxicated with combined exposure to FIP and buprofezin^55^. Moreover, β-glucan recuperated the growth of FIP-intoxicated Nile tilapia^30^. To discuss this point in-depth, it was reported that the growth-stimulating impacts of dietary MO could be accredited to several reasons, such as (a) the presence of essential nutrients such as vitamins (vitamin C, alpha-tocopherol, and vitamin A), minerals (iron, potassium, and iron), and high-quality CP^8^, (b) a plentiful amount of flavonol glycosides (kaempferol and quercetin), and alkaloids (moringinine) in MO leaves, which might enhance the voluntary feed intake via controlling glucose homeostasis^56^, (c) reports also showed that MO leaf powder noticeably enhanced the intestinal morphometry (especially the intestinal villi length) which may be linked with improved growth^52^, and (d) polyphenols such as phenolic acids and flavonoids with prominent growth-stimulating effects^57^.

Regarding the fish body composition analysis, it was found that moisture and CP were considerably increased, combined with a significant decline of the CL in MO groups in relation to other groups. Arsalan et al.^58^ also found that MO leaf meal increased the CP and CL (%) in rohu’s body. In a similar sense, Ganzon-Naret^59^ found increased CP (%) in the body of barramundi fed on a diet supplied with a 10% MO leaf meal. The highest CP (%) in rohu’s body was found in groups fed on a diet supplemented with 15% MO leaf meal^60,61^. Supplementing fermented MO leaves markedly decreased the CL (%) in the gibel carp’s body^62^. In contrast, Dongmeza et al.^7^ reported that supplementing diets with dehydrated MO methanolic extracts showed no significant in the ash, moisture, and CP (%) of Nile tilapia than in the CTR group. These discrepancies may be accredited to fish species, dietary inclusion dose, or MO inclusion forms.

The values of Hb levels, erythrocytes, leukocyte count, and blood protein were significantly lowered in the FIP-intoxicated group. These results proposed the occurrence of anemia and immune suppression. Moreover, liver biomarkers such as ALT and AST enzymes and kidney indices such as blood urea nitrogen and creatinine levels significantly increased in the FIP-intoxicated group. These results suggest the occurrence of hepatotoxicity, liver dysfunction, and hepato-renal damage in the FIP-exposed fish^63,64^. In a similar concern, Gupta et al.^65^ found decreased Hb and serum TP contents alongside a significant increase of AST levels in common carp intoxicated with sub-lethal FIP. Hb levels and leukocyte count were considerably decreased alongside increased serum ALT and AST levels in FIP-intoxicated Nile tilapia^29^. Hb, erythrocyte count, TP, and globulin values were also decreased in FIP-intoxicated common carp^33^. Similarly, exposure of common carp to sub-lethal FIP toxicity resulted in a noticeable decrease in erythrocyte count, Hb, and hematocrit values combined with increased blood urea and creatinine levels^32^. ALT and AST enzyme activities were also increased in the serum of rohu exposed to sub-lethal FIP toxicity^34^. On the other side, it was noticed that supplementing diets with MO leaves extract in FIP-intoxicated groups significantly modulated the aforementioned parameters compared with FIP intoxicated group alone. Mbokane and Moyo^66^ illustrated that supplementing diets with MO leaves essential oil significantly increased packed cell volume, Hb, erythrocyte count, and leukocyte counts of African catfish compared to the CTR group. Serum uric acid, ALT, creatinine, AST, and urea were declined in Nile tilapia fed on moringa leaves meal up to 1.5% than the CTR group^67^. Elabd et al.^17^ illuminated that supplementing diets with MO leaf meal increased the PCV, Hb, erythrocyte count, and leukocyte count than the CTR fish. Also, those authors found a noticeable decline in AST and ALT levels than the CTR group. Furthermore, Monir and coauthors^19^ declared that Nile tilapia fed on MO-supplied diets had significantly elevated TP and GLO concentrations combined with a significant decline in urea, ALT, creatinine, and AST contents. An increase in the PCV, erythrocytes, and leukocyte counts, Hb, and hematocrit levels were recorded in Mozambique tilapia fed on moringa diets^68^. Similarly, Adeshina et al.^69^ found that PCV, Hb, erythrocytes, leukocytes, AST, and ALT values were improved in common carp-fed MO leaves meals. Recently, Abdelkhalek et al.^70^ found that dietary curcumin could alleviate the increased AST and ALT levels in FIP-intoxicated monosex tilapia.

TC and TG levels were increased in the FIP-intoxicated Nile tilapia. TG and TC values were also increased in FIP-intoxicated common carp^32^. However, HDL-c, TG, LDL-c, TC, and VLDL-c values were significantly decreased in MO groups. Monir et al.^19^ described a noticeable decline in serum TG and TC values that Nile tilapia fed on MO-based diets. Furthermore, a noticeable reduction of TC, TG, and LDL-c values was recorded in Nile tilapia fed 5% and 10% MO leaf powder^52^. The reduced serum TC levels may be due to the presence of β-sitosterol^57^, which decreases the absorption of endogenous cholesterol and enhance its secretion from the gastrointestinal tract and excretion as neutral steroids^71^. Moreover, reduced lipoprotein levels (especially LDL-c values) may inhibit cholesterol synthesis, causing the hepatic intracellular sterols to be diminished^71^.

The IgM, complement C3, and LZ levels were decreased in the FIP-intoxicated group. These results suggest that FIP toxicity induced immune suppression in the exposed fish. These findings were parallel to those recorded by El-Murr et al.^29^ and El-Murr et al.^30^, who revealed a considerable decline in LZ and IgM values in the FIP-intoxicated tilapia. A similar decrease in LZ levels was also noticed in monosex Nile tilapia intoxicated with sub-lethal FIP level^70^. Alternatively, the total IgM, complement C3, and LZ were elevated in all MO groups in relation to the FIP and CTR groups. These results were harmonious with those obtained by Mansour et al.^72^, who noticed that diets supplied with 5% MO leaves led to an increment of LZ, and total IgM in gilthead seabream. Moreover, dietary fermented MO leaves improved the immunity biomarkers in gibel carps, especially LZ, IgM levels, and complement component 3 levels^62^. Furthermore, Abd El-Gawad and coauthors^73^ illustrated that MO leaf powder increased LZ activities and IgM of Nile tilapia. The potentiation of the non-specific immune indices of fish fed on MO meals may be accredited to various factors. For example (a) the great number of phytochemicals as polyphenols, carotenoids, and vitamins which possess immune-stimulating functions^74,75^, (b) the secondary metabolites present in MO leaf extract as phenolics (caffeic, p-coumaric, ellagic, protocatechuic, ferulic, and salicylic acids), flavonoids (kaempferol, rutin, quercetin, catechin, apigenin, epicatechin, isorhamnetin, and myricetin), saponins, carotenoids, tannins, alkaloids, terpenoids, β-sitosterol, coumarins, anthocyanins, anthraquinones, and proanthocyanins^9,76^, which are helpful to provoke the fish immune responses, and (c) isothiocyanate can positively contribute to the enhancement of the fish immunity^77^.

Oxidative stress is an overproduction of radicals as reactive oxygen species (ROS), and the body's endogenous antioxidant mechanisms could not scavenge the effects of these ROS^78,79^. In fish, oxidative stress occurs due to exposure to pollutants and xenobiotics^80,81^. The existing study found a meaningful reduction in serum SOD and CAT enzyme activities and T-AOC levels and a substantial rise of MDA concentrations in the FIP-intoxicated group. Similarly, it was found that FIP induced oxidative stress damage in the exposed common carp and silver catfish^27,82^. Moreover, Ghazanfar et al.^55^ described a noticeable reduction in SOD and CAT enzymes in the gills, liver, kidney, and brain of FIP-intoxicated common carp. Furthermore, FIP toxicity induced a decrease in SOD, glutathione peroxidase (GPX), and CAT enzymes and increased MDA contents in the gills and hepatic tissues of rainbow trout^36,37^. Interestingly, Abdelkhalek et al.^70^ also found FIP toxicity is characterized by elevated MDA and NO levels in renal and hepatic tissues of monosex tilapia. Herein, we found that MO leaves extract significantly elevated SOD and CAT enzyme activities and T-AOC levels alongside a considerable decrease of MDA compared with the FIP group. These findings were in harmony with Elabd et al.^17^, who found that MO leaf meal elevated GPX, SOD, and CAT enzymes in Nile tilapia exposed to stress of starvation. In the same sense, supplementing diets with MO leaf extract efficiently modulated pendimethalin-induced oxidative stress injury in Nile tilapia via a substantial elevation of hepatic GPX, SOD, CAT activities, and T-AOC levels as well as a decrease of hepatic MDA levels of the treated fish^41^. Moreover, supplementing diets with 7% MO seeds modulated chlorpyrifos-caused oxidative stress in Nile tilapia via increased hepatic CAT and SOD levels and decreased hepatic MDA contents in treated fish^40^. Dietary supplementation with MO leaf ethanolic extracts significantly counteracted the oxidative stress caused by sub-chronic toxicity by sodium fluoride in Nile tilapia^83^. Recently, MO leaves meals substantially elevated SOD, CAT, GPX enzymes and decreased MDA concentrations in Nile tilapia^19,73^. Several reports also showed that herbal supplements could modulate the antioxidant status of fish. For example, Khafaga et al.^64^ illustrated that supplementing diets with *Origanum vulgare* essential oil ameliorated cypermethrin-caused oxidative stress in common carp. Similarly, dietary curcumin considerably alleviated FIP-induced tissue oxidative stress in monosex tilapia^70^. The antioxidative effects of MO are associated with the presence of ascorbic acid, carotenoids, flavonoids, and vitamin E^84^. Moreover, the relatively plentiful amounts of phenolics can confer the greatest antioxidant effects by stabilizing the free radicals in cells via donation or accepting the electrons^6^. The presence of glucosinolates also plays a pivotal role in increasing the fish's antioxidant abilities^85^.

## Conclusions

In summary, it can be concluded that long-term exposure to a sub-lethal FIP dose caused growth retardation, changes in the body composition, haemato-biochemical changes, immune depression, and oxidative stress of Nile tilapia. Moreover, using MO leaf ethanolic extract as a natural feed supplement can be considered an important herbal phyto-additive for its protecting functions against the negative impacts of sub-lethal FIP. Endorsements for regular supplementation of MO ethanolic extract at a dose rate of 1.0 g/kg as prophylaxis in aquafeed are important for improving the general health of fish against serious toxicological impacts of some emerging aquatic pollutants.

## Supplementary Information

Supplementary Table S1.

## Data Availability

The datasets used and/or analyzed during the current study available from the corresponding author on reasonable request.

## References

[1] Falowo AB (2018). Multi-functional application of *Moringa*
*oleifera* Lam. in nutrition and animal food products: A review. Food Res. Int..

[2] Abd Rani NZ, Husain K, Kumolosasi E (2018). *Moringa* genus: A review of phytochemistry and pharmacology. Front. Pharmacol..

[3] Cheenpracha S (2010). Potential anti-inflammatory phenolic glycosides from the medicinal plant *Moringa oleifera* fruits. Bioorg. Med. Chem..

[4] Khalil SR (2020). *Moringa oleifera* leaves ethanolic extract influences DNA damage signaling pathways to protect liver tissue from cobalt-triggered apoptosis in rats. Ecotoxicol. Environ. Saf..

[5] Nepolean P, Anitha J, Emilin R (2009). Isolation, analysis and identification of phytochemicals of antimicrobial activity of *Moringa oleifera* Lam. Curr. Biotica.

[6] Toppo R, Roy BK, Gora RH, Baxla SL, Kumar P (2015). Hepatoprotective activity of *Moringa oleifera* against cadmium toxicity in rats. Vet. World.

[7] Dongmeza E, Siddhuraju P, Francis G, Becker K (2006). Effects of dehydrated methanol extracts of Moringa (*Moringa*
*oleifera* Lam.) leaves and three of its fractions on growth performance and feed nutrient assimilation in Nile tilapia (*Oreochromis*
*niloticus* (L.)). Aquaculture.

[8] Makkar H, Becker K (1997). Nutritional value and antinutritional components in different parts of *Moringa oleifera* tree. J. Agric. Sci. Camb..

[9] Kou X, Li B, Olayanju JB, Drake JM, Chen N (2018). Nutraceutical or pharmacological potential of *Moringa oleifera* Lam. Nutrients.

[10] Ahmadifar E (2020). Benefits of dietary polyphenols and polyphenol-rich additives to aquatic animal health: An overview. Rev. Fish. Sci. Aquac..

[11] Wapi C (2013). Physico-chemical shelf-life indicators of meat from broilers given *Moringa oleifera* leaf meal. S. Afr. J. Anim. Sci..

[12] Mendieta-Araica B, Spörndly R, Reyes-Sánchez N, Spörndly E (2011). Moringa (*Moringa oleifera*) leaf meal as a source of protein in locally produced concentrates for dairy cows fed low protein diets in tropical areas. Livest. Sci..

[13] Qwele K (2013). Chemical composition, fatty acid content and antioxidant potential of meat from goats supplemented with Moringa (*Moringa oleifera*) leaves, sunflower cake and grass hay. Meat Sci..

[14] Abdel-Latif HMR, Abdel-Daim MM, Shukry M, Nowosad J, Kucharczyk D (2022). Benefits and applications of Moringa oleifera as a plant protein source in Aquafeed: A review. Aquaculture.

[15] El-Son MAM, Hendam BM, Nofal MI, Abdel-Latif HMR (2022). Effects of *Moringa oleifera*-based diets on growth, immunological responses, liver antioxidant biomarkers and expression of immune-related genes in Nile tilapia (*Oreochromis **niloticus*) raised in hapa-in-pond system. Aquac. Res..

[16] Hussain SM (2018). Replacement of fish meal with *Moringa*
*oleifera* leaf meal (MOLM) and its effect on growth performance and nutrient digestibility in *Labeo*
*rohita* fingerlings. Pak. J. Zool..

[17] Elabd H, Soror E, El-Asely A, El-Gawad EA, Abbass A (2019). Dietary supplementation of Moringa leaf meal for Nile tilapia *Oreochromis*
*niloticus*: Effect on growth and stress indices. Egypt. J. Aquat. Res..

[18] Puycha K (2017). Effect of moringa (*Moringa*
*oleifera*) leaf supplementation on growth performance and feed utilization of Bocourti's catfish (*Pangasius*
*bocourti*). Agric. Nat. Resour..

[19] Monir W, Abdel-Rahman MA, El-Din Hassan S, Mansour ES, Awad SMM (2020). Pomegranate peel and moringa-based diets enhanced biochemical and immune parameters of Nile tilapia against bacterial infection by *Aeromonas*
*hydrophila*. Microb. Pathog..

[20] Kaleo IV (2019). Effects of *Moringa*
*oleifera* leaf extract on growth performance, physiological and immune response, and related immune gene expression of *Macrobrachium*
*rosenbergii* with *Vibrio*
*anguillarum* and ammonia stress. Fish Shellfish Immunol..

[21] Tingle CCD, Rother JA, Dewhurst CF, Lauer S, King WJ, Ware GW (2003). Reviews of Environmental Contamination and Toxicology: Continuation of Residue Reviews.

[22] Wang X (2016). Fipronil insecticide toxicology: Oxidative stress and metabolism. Crit. Rev. Toxicol..

[23] Simon-Delso N (2015). Systemic insecticides (neonicotinoids and fipronil): Trends, uses, mode of action and metabolites. Environ. Sci. Pollut. Res..

[24] Schlenk D (2001). Toxicity of fipronil and its degradation products to *Procambarus* sp.: Field and laboratory studies. Arch. Environ. Contam. Toxicol..

[25] Gan J, Bondarenko S, Oki L, Haver D, Li JX (2012). Occurrence of fipronil and its biologically active derivatives in urban residential runoff. Environ. Sci. Technol..

[26] Pisa LW (2015). Effects of neonicotinoids and fipronil on non-target invertebrates. Environ. Sci. Pollut. Res..

[27] Clasen B (2012). Effects of the commercial formulation containing fipronil on the non-target organism *Cyprinus*
*carpio*: Implications for rice-fish cultivation. Ecotoxicol. Environ. Saf..

[28] SantillánDeiú A, Miglioranza KSB, Ondarza PM, de la Torre FR (2021). Exposure to environmental concentrations of fipronil induces biochemical changes on a neotropical freshwater fish. Environ. Sci. Pollut. Res..

[29] El-Murr A, Imam T, Hakim Y, Ghonimi W (2015). Histopathological, immunological, hematological and biochemical effects of fipronil on Nile tilapia (*Oreochromis*
*niloticus*). Vet. Sci. Technol..

[30] El-Murr AEI, Abd El Hakim Y, Neamat-Allah ANF, Baeshen M, Ali HA (2019). Immune-protective, antioxidant and relative genes expression impacts of β-glucan against fipronil toxicity in Nile tilapia, *Oreochromis*
*niloticus*. Fish Shellfish Immunol..

[31] Wagner SD (2017). Developmental effects of fipronil on Japanese Medaka (*Oryzias*
*latipes*) embryos. Chemosphere.

[32] Ghaffar A (2018). Fipronil (Phenylpyrazole) induces hemato-biochemical, histological and genetic damage at low doses in common carp, *Cyprinus*
*carpio* (Linnaeus, 1758). Ecotoxicology.

[33] Qureshi IZ, Bibi A, Shahid S, Ghazanfar M (2016). Exposure to sub-acute doses of fipronil and buprofezin in combination or alone induces biochemical, hematological, histopathological and genotoxic damage in common carp (*Cyprinus*
*carpio* L.). Aquat. Toxicol..

[34] Ghaffar A (2019). Assessment of genotoxic and pathologic potentials of fipronil insecticide in *Labeo*
*rohita* (Hamilton, 1822). Toxin Rev..

[35] Dallarés S (2020). Multibiomarker approach to fipronil exposure in the fish *Dicentrarchus*
*labrax* under two temperature regimes. Aquat. Toxicol..

[36] Uçar A (2021). Assesment of hematotoxic, oxidative and genotoxic damage potentials of fipronil in rainbow trout *Oncorhynchus*
*mykiss*, Walbaum. Toxicol. Mech. Methods.

[37] Uçar A (2020). Determination of Fipronil toxicity by different biomarkers in gill and liver tissue of rainbow trout (*Oncorhynchus*
*mykiss*). In Vitro Cell. Dev. Biol. Anim..

[38] Noureen A (2018). Ameliorative effects of *Moringa*
*oleifera* on copper nanoparticle induced toxicity in *Cyprinus*
*carpio* assessed by histology and oxidative stress markers. Nanotechnology.

[39] Mansour AT (2020). Dietary supplementation of drumstick tree, *Moringa*
*oleifera*, improves mucosal immune response in skin and gills of seabream, *Sparus*
*aurata*, and attenuates the effect of hydrogen peroxide exposure. Fish Physiol. Biochem..

[40] Ibrahim RE, El-Houseiny W, Behairy A, Mansour MF, Abd-Elhakim YM (2019). Ameliorative effects of *Moringa*
*oleifera* seeds and leaves on chlorpyrifos-induced growth retardation, immune suppression, oxidative stress, and DNA damage in *Oreochromis*
*niloticus*. Aquaculture.

[41] Hamed HS, El-Sayed YS (2019). Antioxidant activities of Moringa oleifera leaf extract against pendimethalin-induced oxidative stress and genotoxicity in Nile tilapia, *Oreochromis*
*niloticus* (L.). Fish Phys. Biochem..

[42] Abdelhiee EY (2021). The impact of *Moringa*
*oleifera* on the health status of Nile tilapia exposed to aflatoxicosis. Aquaculture.

[43] Okechukwu PU (2013). Phytochemical and acute toxicity studies of *Moringa*
*oleifera* ethanol leaf extract. Int. J. Life Sci. Biotechnol. Pharma Res..

[44] Zhao X, Wexler P (2005). Encyclopedia of Toxicology.

[45] Percie du Sert N (2020). Reporting animal research: Explanation and elaboration for the ARRIVE guidelines 20. PLoS Biol..

[46] Abdel-Latif HMR (2022). *Astragalus **membranaceus* extract (AME) enhances growth, digestive enzymes, antioxidant capacity, and immunity of *Pangasianodon*
*hypophthalmus* juveniles. Fishes.

[47] Abdel-Latif HMR (2020). The influence of raffinose on the growth performance, oxidative status, and immunity in Nile tilapia (*Oreochromis*
*niloticus*). Aquac. Rep..

[48] Abdel-Latif HMR, Chaklader MR, Shukry M, Ahmed HA, Khallaf MA (2023). A multispecies probiotic modulates growth, digestive enzymes, immunity, hepatic antioxidant activity, and disease resistance of *Pangasianodon*
*hypophthalmus* fingerlings. Aquaculture.

[49] AOAC (2007). Method 2007-04. Association of Official Analytical Chemists. Washington, DC.

[50] Collier HB (1944). Standardization of blood haemoglobin determinations. Can. Med. Assoc. J..

[51] Ellis AE, Stolen JS, Fletcher TC, Rowley AF (1990). Techniques in Fish Immunology.

[52] El-Kassas S (2020). Growth performance, serum lipid profile, intestinal morphometry, and growth and lipid indicator gene expression analysis of mono-sex Nile tilapia fed *Moringa*
*oleifera* leaf powder. Aquac. Rep..

[53] Hu L (2017). Use of fish species from different trophic levels to control algae and water quality: An enclosure experiment in eutrophic area of Xiaojiang River. PLoS One.

[54] Sweilum MA (2006). Effect of sublethal toxicity of some pesticides on growth parameters, haematological properties and total production of Nile tilapia (*Oreochromis*
*niloticus* L.) and water quality of ponds. Aquac. Res..

[55] Ghazanfar M, Shahid S, Qureshi IZ (2018). Vitamin C attenuates biochemical and genotoxic damage in common carp (*Cyprinus*
*carpio*) upon joint exposure to combined toxic doses of fipronil and buprofezin insecticides. Aquat. Toxicol..

[56] Mbikay M (2012). Therapeutic potential of *Moringa*
*oleifera* leaves in chronic hyperglycemia and dyslipidemia: A review. Front. Pharmacol..

[57] Vergara-Jimenez M, Almatrafi MM, Fernandez ML (2017). Bioactive components in *Moringa*
*oleifera* leaves protect against chronic disease. Antioxidants.

[58] Arsalan M (2016). Effects of *Moringa*
*oleifera* leaf meal (MOLM) based diets on carcass composition and hematology of *Labeo*
*rohita* fingerlings. J. Biodivers. Environ. Sci..

[59] Ganzon-Naret ES (2014). Utilization of *Moringa*
*oleifera* leaf meals as plant protein sources at different inclusion levels in fish meal based diets fed to *Lates*
*calcarifer*. Anim. Biol. Anim. Husb..

[60] Imran M (2018). Moringa meal as an alternate protein source for *Labeo*
*rohita* fingerlings: Effects on growth and body composition. Pak. J. Zool..

[61] Mehdi H (2016). Effect of *Moringa*
*oleifera* meal on the growth, body composition and nutrient digestibility of *Labeo*
*rohita*. Int. J. Biosci..

[62] Zhang X (2020). Effects of dietary fish meal replacement by fermented moringa (*Moringa*
*oleifera* Lam.) leaves on growth performance, nonspecific immunity and disease resistance against Aeromonas hydrophila in juvenile gibel carp (*Carassius*
*auratus* gibelio var. CAS III). Fish Shellfish Immunol..

[63] Dawood MAO, Abdel-Tawwab M, Abdel-Latif HMR (2020). Lycopene reduces the impacts of aquatic environmental pollutants and physical stressors in fish. Rev. Aquac..

[64] Khafaga AF, Naiel MAE, Dawood MAO, Abdel-Latif HMR (2020). Dietary *Origanum*
*vulgare* essential oil attenuates cypermethrin-induced biochemical changes, oxidative stress, histopathological alterations, apoptosis, and reduces DNA damage in Common carp (*Cyprinus*
*carpio*). Aquat. Toxicol..

[65] Gupta SK (2014). Haemato-biochemical responses in *Cyprinus*
*carpio* (Linnaeus, 1758) fry exposed to sub-lethal concentration of a phenylpyrazole insecticide, fipronil. Proc. Natl. Acad. Sci. India Sect. B Biol. Sci..

[66] Mbokane EM, Moyo NAG (2020). Effects of dietary levels of essential oil extracts from *Moringa*
*oleifera* and *Artemisia*
*afra* on kidney histology, haemato-immunological parameters and disease resistance in *Clarias*
*gariepinus*. Aquac. Res..

[67] Salama F, Labib L, Al-Sheikh H, Tonsy H, Zaki M (2016). Effect of Moringa, Moringa oleifera, leaves supplementation as a growth promoter on growth performance, body composition, and physiological profile of Nile tilapia *Oreochromis*
*niloticus* (L.) fry. J. Egypt. Acad. Environ. Dev..

[68] Mbokane EM, Moyo NAG (2018). Alterations of haemato-biochemical parameters pre and post-challenge with *Aeromonas*
*hydrophila* and survival of *Oreochromis*
*mossambicus* fed *Moringa*
*oleifera*-based diets. Fish Shellfish Immunol..

[69] Adeshina I, Sani RA, Adewale YA, Tiamiyu LO, Umma SB (2018). Effects of dietary *Moringa*
*oleifera* leaf meal as a replacement for soybean meal on growth, body composition and health status in *Cyprinus*
*carpio* juveniles. Croat. J. Fish..

[70] Abdelkhalek N (2021). Immunological and antioxidant role of curcumin in ameliorating fipronil toxicity in Nile tilapia (*Oreochromis*
*niloticus*). Aquac. Res..

[71] Mehta K, Balaraman R, Amin AH, Bafna PA, Gulati OD (2003). Effect of fruits of *Moringa*
*oleifera* on the lipid profile of normal and hypercholesterolaemic rabbits. J. Ethnopharmacol..

[72] Mansour AT (2018). Effects of dietary inclusion of *Moringa*
*oleifera* leaves on growth and some systemic and mucosal immune parameters of seabream. Fish Physiol. Biochem..

[73] Abd El-Gawad EA, El Asely AM, Soror EI, Abbass AA, Austin B (2020). Effect of dietary *Moringa*
*oleifera* leaf on the immune response and control of *Aeromonas*
*hydrophila* infection in Nile tilapia (*Oreochromis*
*niloticus*) fry. Aquac. Int..

[74] Anwar F, Latif S, Ashraf M, Gilani AH (2007). *Moringa*
*oleifera*: A food plant with multiple medicinal uses. Phytother. Res..

[75] Makkar H, Francis G, Becker K (2007). Bioactivity of phytochemicals in some lesser-known plants and their effects and potential applications in livestock and aquaculture production systems. Animal.

[76] Wang L, Chen X, Wu A (2016). Mini review on antimicrobial activity and bioactive compounds of *Moringa*
*oleifera*. Med. Chem..

[77] Sudha P, Asdaq S, Dhamingi SS, Chandrakala GK (2010). Immunomodulatory activity of methanolic leaf extract of *Moringa*
*oleifera* in animals. Indian J. Physiol. Pharmacol..

[78] Le Bras M, Clement MV, Pervaiz S, Brenner C (2005). Reactive oxygen species and the mitochondrial signaling pathway of cell death. Histol. Histopathol..

[79] Nordberg J, Arnér ESJ (2001). Reactive oxygen species, antioxidants, and the mammalian thioredoxin system. Free Radic. Biol. Med..

[80] Chowdhury S, Saikia S (2020). Oxidative stress in fish: A review. J. Sci. Res..

[81] Lackner R, Braunbeck T, Hinton DE, Streit B (1998). Fish Ecotoxicology.

[82] Menezes C (2016). Effect of diphenyl diselenide diet supplementation on oxidative stress biomarkers in two species of freshwater fish exposed to the insecticide fipronil. Fish Physiol. Biochem..

[83] Ahmed NF (2020). *Moringa*
*oleifera* leaf extract repairs the oxidative misbalance following sub-chronic exposure to sodium fluoride in Nile tilapia *Oreochromis*
*niloticus*. Animals.

[84] Pakade V, Cukrowska E, Chimuka L (2013). Comparison of antioxidant activity of *Moringa*
*oleifera* and selected vegetables in South Africa. S. Afr. J. Sci..

[85] Bennett, R. N. *et**al.* Profiling glucosinolates and phenolics in vegetative and reproductive tissues of the multi-purpose trees *Moringa**oleifera* L. (Horseradish Tree) and *Moringa**stenopetala* L. *J.**Agric.**Food**Chem.* **51**, 3546–3553. 10.1021/jf0211480 (2003). 10.1021/jf0211480 12769522

