# Supplementary material for: Dietary supplementation with Moringa oleifera leaves extract reduces the impacts of sub-lethal fipronil in Nile tilapia, Oreochromis niloticus
Source: Sci Rep. 2022 Dec 16;12:21748. doi: 10.1038/s41598-022-25611-6 (PMC9758223; doi:10.1038/s41598-022-25611-6)
Supplement: Supplementary file 1 — Supplementary Table S1. [file 41598_2022_25611_MOESM1_ESM.docx]

**Supplementary materials**

**Table S1**

Feed ingredients and proximate chemical composition analysis of the basal commercial diet (dry weight, %) used in the present study.

| **Feed ingredients** | **%** |
| --- | --- |
| Fish meal (72%) | 6 |
| Soybean meal (46%) | 33 |
| Corn gluten (60%) | 14 |
| Yellow corn | 15.5 |
| Wheat bran | 20 |
| Vegetable oil | 2 |
| Fish oil | 2 |
| Starch | 5 |
| Lysine | 0.5 |
| Mineral premix ^a^ | 1 |
| Vitamin premix ^b^ | 1 |
| Total | 100 |
| **Proximate composition (%) as fed basis** | |
| Crude protein (CP) | 30.76 |
| Ether extract (EE) | 6.98 |
| Crude fiber (CF) | 6.55 |
| Ash | 7.24 |
| Nitrogen free extract (NFE) ^c^ | 48.47 |

^a^ Mineral premix (per kg of premix): Calcium carbonate as carrier up to 1 kg for zinc,40 g; iron, 20 g; copper, 2.7 g; iodine, 0.34 g; manganese,53 g; selenium, 70 mg and cobalt, 70 mg

^b^ Vitamin premix (per kg of premix): Vitamin B_1_, 700 mg; Vitamin B_2_, 3500 mg; Vitamin B_6_, 1000 mg; Vitamin B_12_, 7 mg; Vitamin A,8000000 IU; Vitamin D_3_, 2000000 IU; Vitamin E, 7000 mg; Vitamin K_3_,1500 mg; biotin, 50 mg; folic acid, 700 mg; nicotinic, 20000 mg; pantothenic acid,7000 mg

^c^ NFE = 100- (CP%+ EE%+ CF%+ Ash%)]
